# HemPepPred: Quantitative Prediction of Peptide Hemolytic Activity Based on Machine Learning and Protein Language Model–Derived Features

**DOI:** 10.3390/foods14234143

**Published:** 2025-12-03

**Authors:** Xiang Li, Wanting Zhao, Xiao Liang, Xinlan Zhuo, Shuang Yu, Guizhao Liang

**Affiliations:** Key Laboratory of Biorheological Science and Technology, Ministry of Education, College of Bioengineering, Chongqing University, Chongqing 400044, China; 202319021049t@stu.cqu.edu.cn (X.L.); 202419021001@stu.cqu.edu.cn (W.Z.); 20241901014@stu.cqu.edu.cn (X.L.); 202319131087t@stu.cqu.edu.cn (X.Z.); 202319131159@stu.cqu.edu.cn (S.Y.)

**Keywords:** hemolytic peptide, ensemble learning

## Abstract

Accurate prediction of hemolytic peptides is essential for peptide safety evaluation and therapeutic design; however, existing models remain constrained by limited accuracy and interpretability. To overcome these challenges, we propose a regression framework that integrates embeddings from a protein language model with handcrafted amino acid descriptors. Specifically, sequence representations derived from the ESM2_t33 model are fused with physicochemical amino acid descriptor features, and key predictive variables are selected through a three-stage strategy involving variance filtering, F-test ranking, and mutual information analysis. The final ensemble model, composed of Random Forest, Extremely Randomized Trees, Gradient Boosting, eXtreme Gradient Boosting (XGBoost), and Ridge Regression, achieved a coefficient of determination (R^2^) of 0.57 and a correlation coefficient (R) of 0.76 on the test set, outperforming previous approaches. To enhance interpretability, we applied Shapley value analysis and the Calibrated_Explanation algorithm to quantify feature contributions and generate reliable sample-specific explanations. The trained model has been deployed online as HemPepPred, a tool for predicting hemolytic concentration (HC_50_) values, which provides a practical platform for rational peptide design and safety assessment.

## 1. Introduction

Food-derived bioactive peptides (BAPs) have emerged as promising ingredients of functional foods and nutraceuticals for health and preventing disease [1]. Extensive research has demonstrated that these peptides exhibit diverse biological activities, including antihypertensive, antimicrobial, anticoagulant, anticancer, anti-inflammatory, and antidiabetic effects [2]. Despite their considerable potential in the food industry and clinical nutrition, the possible toxicity of BAPs remains a major obstacle to large-scale application.

Under certain conditions, some peptides can misfold and form β-sheet aggregates similar to human islet amyloid polypeptides (hIAPPs) associated with type 2 diabetes, leading to disruption of cell membrane integrity and subsequent cytotoxicity [3]. This process mirrors the oxidative damage mechanisms observed with β-amyloid in Alzheimer’s disease [4]. The occurrence of toxic peptides in a wide variety of food sources has long been recognized, posing significant challenges to food safety and industry growth. A well-known example is the 33-mer proline-rich peptide derived from gluten proteins in wheat and barley, which acts as a major immunogenic epitope in celiac disease—a hereditary autoimmune disorder affecting approximately 1% of the global population [5]. Other plant-derived foods also contain potentially harmful peptides. For instance, lectins from legumes that are not adequately degraded during digestion can cause acute lectin poisoning, characterized by nausea, diarrhea, vomiting, and agglutination of red blood cells [6]. Furthermore, the soybean toxin, a single-chain acidic protein, exhibits marked toxicity in mice, inducing tonic–clonic seizures, flaccid paralysis, and ultimately death following injection [7]. Additionally, cassava contains cyanogenic glycosides such as linamarin and lotaustralin, which release the potent toxin hydrogen cyanide during enzymatic hydrolysis, and inadequate processing (e.g., insufficient soaking or fermentation) can therefore result in acute cyanide poisoning [8]. Collectively, these examples underscore the urgent need for the identification and evaluation of peptide toxicity in food systems.

The hemolytic concentration (HC_50_) is a classical indicator of peptide toxicity, defined as the concentration that causes 50% lysis of normal human red blood cells under physiological conditions [9]. Conventional in vitro assays, including the MTT and lactate dehydrogenase (LDH) release tests, can directly measure cytotoxicity but are limited by low throughput and lengthy turnaround times [10]. Consequently, developing efficient and accurate computational approaches has become a key priority in both drug discovery and functional food safety evaluation [11].

Early work primarily addressed the binary classification of peptides as hemolytic or non-hemolytic, whereas more recent approaches have shifted toward quantitative modeling of hemolytic activity, particularly the prediction of hemolytic concentration (HC_50_). Various machine learning strategies have been developed to improve prediction accuracy. Rathore et al. (2025) proposed a hybrid model that achieved an AUC of 0.909 for HC_50_ prediction, with a companion regression model attaining a correlation coefficient of 0.739 [12]. Plisson et al. (2020) reported a gradient boosting classifier that reached 95–97% accuracy and enhanced reliability through outlier detection [13]. In 2024, Yang and Xu introduced HemoDL, which integrates a dual-LightGBM framework with composition-transition-distribution (CTD) and transformer-derived sequence features, outperforming conventional methods in predicting hemolytic activity [14]. Almotair et al. (2024) designed a hybrid architecture combining Transformer and CNN networks, emphasizing that balanced train-test partitioning is essential for robust generalization [15]. Similarly, Raza and Arshad (2020) demonstrated that appropriate dataset partitioning improves predictive accuracy and AUC-ROC performance [16]. Karasev et al. (2024) highlighted the impact of experimental conditions on model precision and reported a regression R^2^ of 0.69 [17]. Additionally, Castillo-Mendieta (2024) developed a multi-query similarity search approach [18], while Yaseen et al. (2021) introduced HemoNet, a neural network model—both methods outperformed traditional machine learning baselines [19]. Hasan et al. (2020) further advanced this field with HLPPred-Fuse, a two-tier framework that integrates multiple feature representations to enhance predictive performance [20].

Recent studies indicate a growing trend toward refined modeling of hemolytic activity; however, high-accuracy regression prediction of HC_50_ remains limited. As model complexity and predictive performance continue to increase, the need for robust interpretability has become more pressing. Notable approaches—including SHapley Additive exPlanations (SHAP) [21], Local Interpretable Model-agnostic Explanations (LIME) [22] and Anchor Explanations (Anchor) [23]—enable feature-importance analysis to elucidate the decision basis underlying specific model outputs and have been applied in hemolytic-peptide regression tasks. Nevertheless, these methods typically provide only point estimates of feature contributions and, therefore, fail to capture the uncertainty associated with both predictions and importance assessments. This limitation reduces their reliability in high-stakes applications, underscoring the need for more resilient interpretability frameworks capable of quantifying feature-level uncertainty for individual samples.

**Figure 1:**
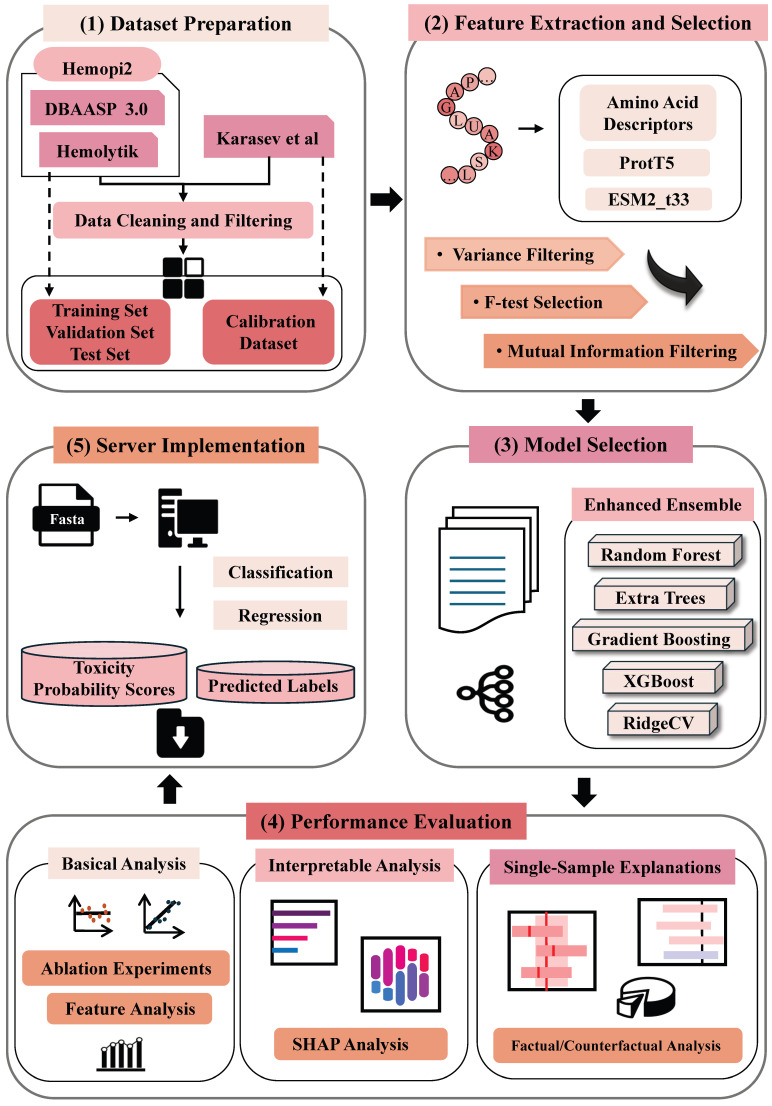
Workflow of Hemolytic Peptide Toxicity Prediction: (1) Dataset Preparation; (2) Feature Extraction and Selection; (3) Model Selection; (4) Performance Evaluation; (5) Server Implementation.

To address this gap, we propose a regression framework for HC_50_ prediction that integrates protein language model embeddings with amino acid descriptors (AAD). In contrast to conventional classification methods that merely discriminate between hemolytic and non-hemolytic peptides, the proposed framework enables fine-grained quantitative prediction of HC_50_, offering higher-resolution guidance for the rational design of hemolytic peptides. The predictor is constructed using an ensemble learning strategy, with global interpretability provided by SHAP [24,25,26]. Furthermore, we incorporate the Calibrated_Explanation algorithm to generate confidence intervals for sample-level feature importance, thereby enhancing the robustness and reliability of model interpretation [27]. Finally, the trained model is deployed as HemPepPred, available online: http://hem.cqudfbp.net (accessed on 22 October 2025), which is an online platform for HC_50_ prediction that provides an efficient and interpretable tool for rational hemolytic peptide design and food safety evaluation (Figure 1).

## 2. Materials and Methods

### 2.1. Dataset

The dataset employed in this study was derived from the Hemopi2 project by Rathore et al., originally compiled for the qualitative and quantitative analysis of hemolytic activity. It comprises 3147 experimentally validated hemolytic peptides from the DBAASP database and 560 peptides from the Hemolytik database [28]. In this work, we directly utilized the curated dataset from Rathore et al., in which peptides containing non-natural amino acids or fewer than six residues had already been excluded as part of the original preprocessing procedure. For peptides reported with multiple HC_50_ values or value ranges, the mean HC_50_ was calculated to represent the overall hemolytic activity under different experimental conditions, thereby improving the robustness of the predictive model. The descriptive statistics of the Hemopi2 dataset are provided in Table 1.

All HC_50_ values were standardized to a consistent unit (µM) and subsequently converted to pHC_50_ values using Equation (1), facilitating normalization and enhancing the model’s capacity to capture relative differences in hemolytic potency.

(1) $$y_{log}=-\log_{10}\left(y+1\times 10^{-8}\right),$$

The calibration dataset, obtained from Karasev et al. [17] in CSV format, comprised peptides with experimentally validated HC_50_ values (µM) and followed the same format as the training and test datasets.

### 2.2. Feature Engineering

Feature engineering constituted the core of constructing an ensemble learning model [29]. This process entailed extracting informative descriptors from peptide sequences and refining them through multi-stage selection.

In this study, three categories of features were utilized to represent peptide sequences. First, we used Pfeature to extract 1167-dimensional classical AAD [30] from the Hemopi2 study’s ALLCOMP-ex SOC results, including AAC, DPC, ATC, and BTC features. These descriptors capture physicochemical and compositional properties known to influence peptide activity. In addition, high-dimensional embeddings from protein language models were incorporated: the ProtT5 model [31] produced 1024-dimensional embeddings encoding deep structural and functional information through global context modeling, while the ESM2_t33 model [32] generated 1280-dimensional vectors capturing evolutionary and structural patterns.

Collectively, these three feature representations complement one another by linking sequence-derived physicochemical characteristics with higher-order structural and contextual information. This integration provides a mechanistic basis for understanding sequence-activity relationships beyond the capacity of traditional descriptors alone. According to Equation (2), the three feature sets were horizontally concatenated using the np.hstack function to construct an initial composite feature matrix containing 3471 features, thereby providing a comprehensive foundation for subsequent feature selection. Because the ensemble primarily consisted of tree-based models, which are invariant to feature scaling, no normalization was applied before concatenation. The Ridge regression component was trained and validated under the same preprocessing to ensure consistency across models.

(2) $$X_{\mathrm{combined}}=\left[X_{aa},X_{m1},X_{m2}\right]\in R^{n\times 3471},$$

### 2.3. Feature Selection

A three-stage strategy was applied for feature selection to progressively reduce dimensionality by eliminating redundant and low-information features. The specific workflow is as follows:

First, variance filtering (threshold < 0.005) was applied to eliminate features with minimal variance, as such features contribute little to discriminative power.

(3) $$\mathrm{Var}\left(x_{j}\right)=\frac{1}{n}\sum_{i=1}^{n}{\left(x_{ij}-\bar{x_{j}}\right)}^{2},\;\bar{x_{j}}=\frac{1}{n}\sum_{i=1}^{n}x_{ij},$$

Next, an F-test (using *f_regression* in the *SelectKBest* method) ranked features according to their linear correlation with the target variable, retaining the top 80% ($k_{f}=0.8\times n_{var}$).

(4) $$F=\frac{MSR}{MSE},\;MSR=\sum_{i=1}^{n}{\left(\hat{y_{i}}-\bar{y}\right)}^{2},\;MSE=\frac{\sum_{i=1}^{n}{\left(y_{i}-\hat{y_{i}}\right)}^{2}}{n-2},$$

Finally, mutual information analysis (*mutual_info_regression*) was used to capture nonlinear dependencies, selecting the top $k_{mi}=0.5\times n_{f}$ features. When the number of selected features exceeded the target range (600–700), a secondary F-test ranking was performed to refine the final subset.

(5) $$I\left(X;Y\right)=\sum_{x\in X}\sum_{y\in Y}p\left(x,y\right)\log\left(\frac{p\left(x,y\right)}{p\left(x\right)p\left(y\right)}\right),$$

To ensure robust model training, we applied tailored preprocessing to the selected features. The feature matrix was first transformed using a QuantileTransformer to mitigate scale bias and capture nonlinear relationships, followed by standardization with RobustScaler to enhance convergence stability. This preprocessing pipeline produced a compact, well-scaled dataset, thereby improving predictive accuracy and computational efficiency in hemolytic peptide regression.

### 2.4. Cross-Validation

Following standard bioinformatics practice, the dataset was randomly partitioned into a training set (80%) and a test set (20%). Model performance was assessed using five-fold cross-validation within the training set. Specifically, the data were randomly divided into five folds, with each iteration using four folds for training and one for validation, ensuring that every fold served as the validation set exactly once. The test set remained completely isolated from all training, validation, and hyperparameter tuning procedures to guarantee an unbiased final evaluation. Additionally, random seeds were fixed throughout all experiments to ensure reproducibility.

### 2.5. Machine Learning Model Construction

An enhanced ensemble framework was employed by integrating multiple regression algorithms, including Random Forest Regressor, Extremely Randomized Trees (Extra Trees) Regressor, Gradient Boosting Regressor, eXtreme Gradient Boosting (XGBoost) Regressor, and Ridge Regression (Ridge CV). The hyperparameter ranges for each model are provided in Supplementary Table S1.

Each model’s five-fold cross-validation score served as its base weight, reflecting its reliability. To enhance ensemble discrimination, a nonlinear transformation (score^2^) was applied to amplify the contribution of consistently strong learners. Furthermore, empirical calibration showed that moderate scaling factors (1.3–2.0×) improved predictive stability and mitigated overfitting tendencies in tree-based models. Specifically, models achieving cross-validation scores above 0.9 and 0.8 were assigned weight multipliers of 2× and 1.5×, respectively, while the Ridge model was upweighted by 1.3× to counterbalance the overfitting tendency of tree-based regressors. Finally, all weights were normalized, and the final prediction was computed as a weighted average.

### 2.6. Performance Evaluation

Model performance was evaluated using five metrics, grouped into two functional categories: (i) goodness-of-fit metrics, including the coefficient of determination (R^2^) and correlation coefficient (R); and (ii) error-based metrics, including mean squared error (MSE), mean absolute error (MAE), and root mean squared error (RMSE). The mathematical formulations of these metrics are shown in Equations (6)–(9):

(6) $$R^{2}=1-\frac{\sum {\left(y_{i}-\hat{y_{i}}\right)}^{2}}{\sum {\left(y_{i}-\bar{y}\right)}^{2}},$$

(7) $$R=\frac{\sum \left(y_{i}-\bar{y}\right)\left(\hat{y_{i}}-\bar{\hat{y}}\right)}{\sqrt{\sum {\left(y_{i}-\bar{y}\right)}^{2}\sum {\left(\hat{y_{i}}-\bar{\hat{y}}\right)}^{2}}},$$

(8) $$MAE=\frac{1}{n}\sum_{i=1}^{n}\left|y_{i}-\hat{y_{i}}\right|,$$

(9) $$RMSE=\sqrt{\frac{1}{n}\sum {\left(y_{i}-\hat{y_{i}}\right)}^{2}}=\sqrt{MSE},$$

### 2.7. Global Model Interpretation

To elucidate the model’s decision process, we employed SHAP, a method grounded in cooperative game theory. SHAP quantifies the contribution of each feature to a prediction by evaluating its marginal impact across all possible feature combinations. Specifically, the algorithm estimates the contribution of a feature to an individual prediction by iteratively adding or removing features, assigning a Shapley value that ensures fair attribution of importance. The underlying principle of Shapley values is to comprehensively assess each feature’s influence by integrating its contributions over all possible feature subsets.

In this study, SHAP values were computed for the entire ensemble model across all test samples, enabling interpretation of the ensemble’s integrated decision boundaries and reflecting the joint effect of its constituent models. We quantified the impact of each feature on model predictions and identified the top 20 most influential features along with their corresponding Shapley values and indices. These features highlight the primary determinants of hemolytic peptide toxicity, offering valuable insights for further model refinement. Additionally, a global feature importance plot was generated using the summary_plot function, illustrating the distribution of Shapley values and providing an intuitive overview of each feature’s overall influence. These analyses enhance the interpretability of the model and serve as a reference for subsequent feature selection and optimization.

### 2.8. Single-Sample Explanations

While SHAP was employed to interpret global feature importance across the ensemble, single-sample explanations and prediction uncertainty were further analyzed using the Calibrated Explanations framework built upon the Conformal Predictive Systems (CPS) methodology. In this framework, SHAP-derived feature attributions were calibrated to provide statistically valid confidence bounds for each individual prediction.

Using the Python (3.10) package calibrated-explanations, we implemented two distinct interpretable modes: standard regression for calibrating continuous HC_50_ values, and probabilistic regression for estimating the probability of HC_50_ exceeding a specific threshold, respectively. The calibration dataset used in this step was derived from Karasev et al. [17], consisting of peptides with experimentally validated HC_50_ values.

## 3. Results and Discussion

### 3.1. Analysis of the Amino Acid Composition

**Figure 2:**
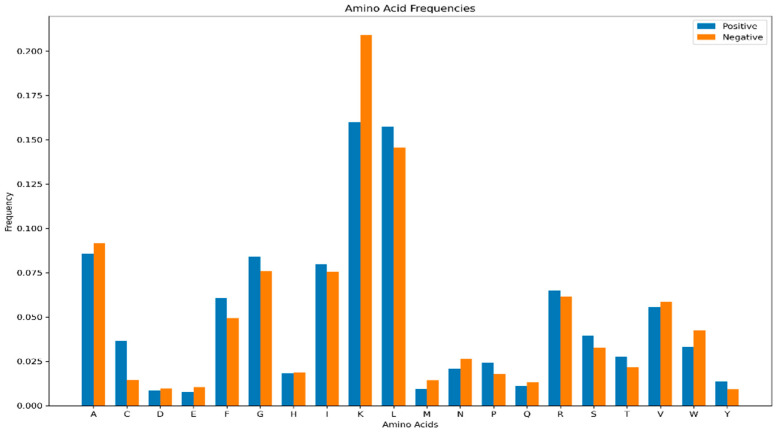
Amino Acid Composition of the Hemopi2 Dataset.

Comparative analysis of amino acid composition revealed distinct distributional patterns between hemolytic and non-hemolytic peptides (Figure 2). Lysine (K) occurred at a significantly higher frequency in non-hemolytic samples, whereas Leucine (L) was notably more abundant in hemolytic peptides. The remaining amino acids exhibited relatively balanced distributions across both groups, with less pronounced differences than those observed for K and L.

These compositional differences suggest underlying physicochemical disparities between hemolytic and non-hemolytic peptides. The abundance of L in hemolytic peptides may enhance hydrophobic interactions with cell membranes, thereby facilitating membrane disruption and hemolytic activity. Conversely, the higher frequency of K in non-hemolytic peptides might reduce overall hydrophobicity or impede membrane binding, ultimately diminishing hemolytic potential. These residue-specific patterns provide a crucial physicochemical foundation for interpreting the feature importance results of the ensemble learning model.

### 3.2. Ablation Experiments

Ablation experiments were conducted to identify key factors influencing model performance. We systematically removed specific feature groups, including AAD, features derived from the large protein language model ProtT5, and embeddings from the ESM2_t33 model. Table 2 summarizes the outcomes and highlights the effectiveness of the feature-selection strategy, showing that AAD and ESM2_t33 embeddings are the two most influential contributors to regression performance.

Feature selection played a decisive role in improving the ensemble model across all experimental settings, regardless of whether individual feature types (AAD, ESM2_t33, or ProtT5) were excluded or combined. For instance, in the “Without AAD” setting, applying feature selection reduced the MAE from 0.3689 to 0.3471 and the RMSE from 0.4779 to 0.4528, while the R^2^ increased from 0.4364 to 0.4942. These results confirm that feature selection enhances both predictive accuracy and generalization capacity.

The further analysis of the outcomes from various feature combinations revealed variations in the efficacy of feature selection among them. The “Without ESM2_t33” experiment demonstrated the most pronounced effect setting and exhibited the most pronounced improvement, where MAE decreased from 0.3636 to 0.3115, RMSE from 0.4729 to 0.4201, and R^2^ increased from 0.4482 to 0.5645. Although excluding ProtT5 features resulted in smaller gains, the combination of manually extracted AAD and ESM2_t33 embeddings achieved the best overall performance across all four evaluation metrics. Accordingly, this feature set was adopted for all subsequent experiments.

Overall, these findings demonstrate that the feature-selection pipeline consistently reduces model error and enhances robustness, with particular efficacy in handling high-dimensional data.

### 3.3. Regression Performance

Given the strong performance of the handcrafted AAD and the ESM2_t33 embeddings, the two feature sets were selected as inputs for the final regression model. Using this combined feature representation, we employed 80% of the Rathore et al. dataset for training and the remaining 20% for testing. Model performance was evaluated by comparing predicted and true −log(HC_50_) values.

**Figure 3:**
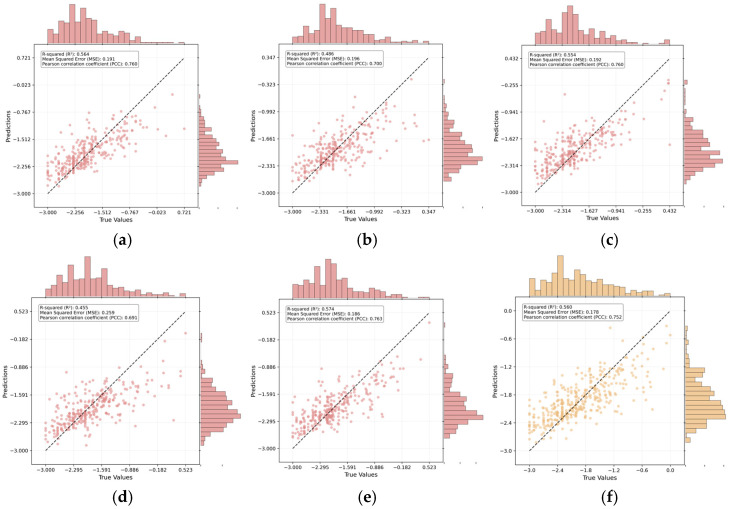
Scatter plot of −log(HC_50_), (**a**–**e**) Scatter plots of predicted versus true −log(HC_50_) values from the five-fold cross-validation on the training set. The solid line in each panel represents the line of perfect prediction (y = x). The corresponding performance metrics (R^2^, MSE, PCC) for each fold are annotated within the plots. (**f**) Scatter plot of predicted versus true -log(HC_50_) values on the independent test set.

As shown in Figure 3, the diagonal distribution of data points suggests a strong agreement between predicted and actual values. Quantitatively, the model achieved an R^2^ of 0.57 and an R of 0.76, confirming reliable predictive capability for hemolytic activity. Model stability was further verified through five-fold cross-validation on the training set, yielding consistent results (average R^2^ ≈ 0.53, average PCC ≈ 0.73). The close alignment between the cross-validation and independent test performance demonstrates the model’s robustness and generalization capability, effectively ruling out overfitting. Moreover, the comparable distributions of predicted and observed values, visualized through histograms, further support this consistency.

Having established the base performance of our model, we performed a comparative analysis against established benchmarks. We compared the performance with eight other machine learning regressors, such as RF, XGBoost (XGB), Decision Tree (DT), Adaptive Boosting (ADB), Support Vector Machine (SVM), K-Nearest Neighbors (KNN) and Extra Trees (ET). As summarized in Table 3, the results revealed clear differences in error control, explanatory power, and correlation strength. ADB achieved the lowest MAE (0.254) and RMSE (0.400), indicating superior error control, but its R^2^ (0.374) and R (0.625) suggested limited explanatory capacity. In contrast, RF and ET demonstrated balanced performance, with R^2^ values around 0.53–0.56 and relatively low errors. Other algorithms, including XGB, DT, SVM, and KNN, lagged behind across all metrics.

The proposed ensemble model achieved the highest R^2^ (0.565) and R (0.755), indicating the strongest fit and linear correlation with experimental data, despite its moderate MAE (0.313) and RMSE (0.420). Collectively, ADBR proves optimal when minimizing prediction error is the primary objective, whereas the proposed model excels in scenarios prioritizing interpretability and predictive correlation. Meanwhile, RF and ET offer a balanced compromise.

**Figure 4:**
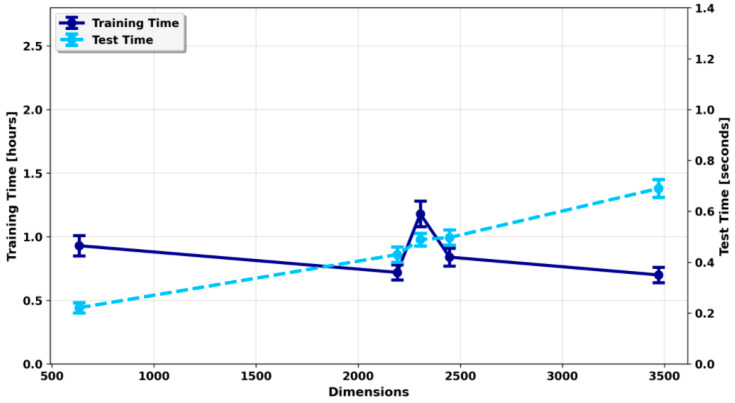
Training and testing time of the ensemble model at different feature dimensions.

To further evaluate practical performance, we investigated computational efficiency under varying dimensional complexity. As model dimensionality increased, testing time rose correspondingly from 0.221 s to 0.690 s (Figure 4), reflecting the higher computational burden of larger feature spaces. Training time exhibited more complex behavior: although it remained relatively stable overall, slight decreases were observed within certain dimensional ranges, likely due to optimization dynamics or hardware utilization differences. In general, testing time was more sensitive to dimensionality, whereas training time was influenced by multiple interacting factors, including model complexity and system performance.

In summary, this study successfully established an effective regression model for hemolytic activity by integrating AAD and ESM2_t33 embeddings. The model demonstrated strong predictive accuracy (R^2^ = 0.57, R = 0.76) and consistently outperformed baseline algorithms across key metrics. Despite the computational demands of high-dimensional input features, the model achieved an effective balance between predictive accuracy, interpretability, and efficiency—making it a practical and reliable tool for quantitative hemolytic activity prediction.

### 3.4. Global Interpretability Analysis

**Figure 5:**
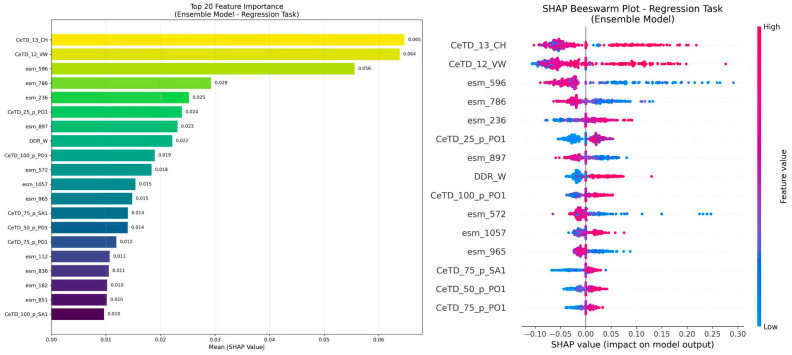
Top 20 features utilized in the regression model. The left bar plot shows the ranked features, with importance calculated by averaging Shapley values over the Sreg test dataset. The right beeswarm plot illustrates the influence of different feature values on the prediction.

To elucidate the determinants of model predictions, SHAP analysis was applied directly to the integrated ensemble model to quantify feature contributions. Figure 5 displays the top 20 most influential features and their corresponding Shapley values. In this study, the sign of the Shapley values directly corresponds to the direction of influence: negative values correspond to lower predicted hemolytic activity, whereas positive values indicate higher predicted activity.

Notably, the most impactful features included both engineered AADs (e.g., CeTD_12_VW, CeTD_13_CH) and ESM2_t33 embeddings (e.g., esm_572, esm_688, esm_897), confirming that these two types of features provide complementary information. The AADs capture interpretable physicochemical trends, while the ESM2_t33 embeddings encode contextual and structural dependencies within sequences. Their integration enables the ensemble to exploit both explicit biochemical knowledge and implicit sequence representations, enhancing predictive robustness and interpretability.

Among these, the most influential feature, CeTD_12_VW, characterizes global sequence composition, transitions, and distributions based on the 12th van der Waals volume grouping. It exhibits a clear monotonic pattern: higher values (red) correspond to positive Shapley values that increase predicted hemolytic activity, whereas lower values (blue) associate with negative Shapley values, reducing the predicted response. This relationship reflects a direct mechanistic linkage between sequence-level physicochemical properties and hemolytic potential.

### 3.5. Single-Sample Interpretability Analysis

To further investigate the model’s decision pathways and quantify predictive uncertainty, we employed Calibrated Explanations based on the Conformal Predictive Systems framework. Using the first test sample as an illustrative case, both factual and counterfactual explanations were generated for standard and probabilistic regression analyses. Table 4 presents the relevant information for the peptide sequence that is subsequently used in the single-sample explanation.

**Figure 6:**
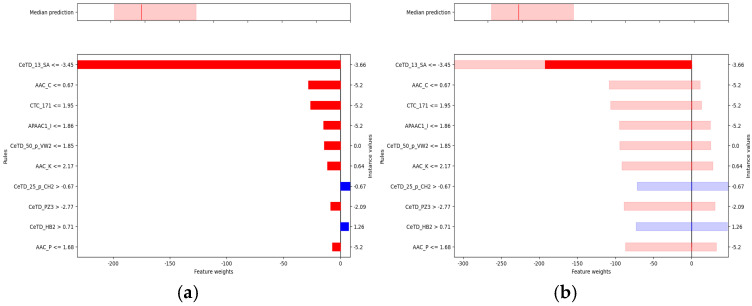
The blue bars indicate positive feature weights that increase the predicted value, and red bars indicate negative weights that decrease it. (**a**) Regular plot for the Hemopi2 dataset. The top subplot displays median values and confidence intervals, while the lower subplot visualizes the feature importance. (**b**) Uncertainty plot for the Hemopi2 dataset. This figure shares the same top subplot as (**a**), while the lower subplot highlights the uncertainty associated with each feature’s weight using shaded percentiles.

In the baseline interpretability analysis of the Hemopi2 dataset (Figure 6a), the model generated a calibrated prediction for the selected test peptide. The upper panel shows a predicted median HC_50_ of approximately 300.9 μM (red line), with the shaded area indicating the 90% confidence interval (5th–95th percentiles). This indicates that the true HC_50_ value is expected to fall within this range. The lower panel illustrates the contributions of key features to the prediction: negative weights (red) represent features that decrease the predicted HC_50_, whereas positive weights (blue) indicate features that increase it.

Among these predictors, the Composition-enhanced Transition and Distribution (CeTD) descriptors exerted the strongest influence. CeTD features integrate amino acid composition, transition probabilities, and positional distributions based on physicochemical classifications. For example, CeTD_13_SA captures the transition and distribution of amino acids grouped by solvent accessibility, describing how residues with different exposure levels are arranged and how these patterns affect peptide-membrane interactions. When CeTD_13_SA ≤ −3.45, its negative effect is maximal, reducing the predicted HC_50_ by nearly 200 μM. Similarly, lower values of AAC_C (the relative abundance of cysteine residues) and CTC_171 (a conjoint triad feature representing short-range residue combinations) are associated with decreased HC_50_, suggesting that diminished cysteine content and specific local residue arrangements may attenuate hemolytic potential.

Conversely, CeTD_25_p_CH2 and CeTD_HB2 exhibit positive contributions. CeTD_25_p_CH2 reflects transitions among residues rich in −CH_2_− side chains, indicating elevated hydrophobic carbon content, while CeTD_HB2 captures higher-order distributions based on hydrogen-bonding potential. When CeTD_25_p_CH2 > −0.67 and CeTD_HB2 > 0.71, both features increase the predicted HC_50_, implying that enhanced hydrophobicity and hydrogen-bonding networks may jointly mitigate hemolytic activity.

Uncertainty analysis for the same Hemopi2 sample (Figure 6b) incorporated predictive confidence into feature attribution. CeTD_13_SA remained the dominant negative factor, with its 90% confidence interval indicating at least a 190 μM reduction in HC_50_ under the specified condition. In contrast, AAC_C and CTC_171 exhibited intervals crossing zero, implying statistically indeterminate directional effects. Because feature normalization was not applied, the interval widths were directly comparable, allowing consistent evaluation of absolute effect magnitudes.

Taken together, these results demonstrate that the model delivers robust point predictions while simultaneously quantifying the confidence of feature contributions, enabling a more comprehensive understanding of the molecular determinants of peptide hemolysis. These findings emphasize that CeTD descriptors, which capture transitions and distributions among amino acid groups, particularly in terms of solvent exposure, hydrophobicity, and hydrogen-bonding potential, are major contributors to hemolytic behavior. This integration of explainable machine learning with physicochemical sequence analysis provides valuable mechanistic insight and a rational basis for subsequent peptide design and experimental validation.

**Figure 7:**
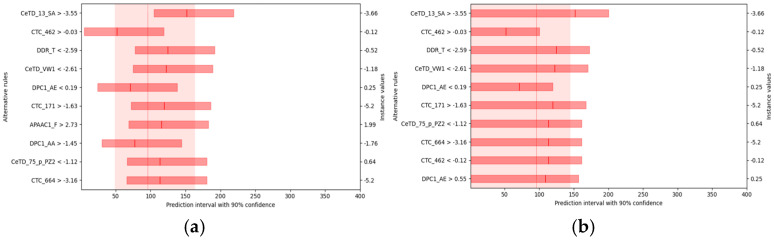
(**a**) The counterfactual plot for the hemolytic peptide dataset. (Each row represents a rule, showing its median (solid line) and 5th–95th percentile confidence interval. The overall background provides the original instance’s confidence interval for reference); (**b**) One-sided counterfactual plot using the 90th upper percentile only to define confidence intervals, demonstrating the effect of conditional violations.

For each rule, the solid line and the lighter red band in the figure represent the expected median and confidence interval of the predicted HC_50_ when the sample satisfies that rule. As shown in Figure 7a, when all other conditions remain constant and the median solvent accessibility exceeds −3.55, the expected HC_50_ is approximately 150 μM. Evidently, a decrease in the amino acid transition ratio (DDR_T) or in the distribution proportion of amino acids grouped by van der Waals volume (CeTD_VW1) results in a higher HC_50_, whereas an increase in these parameters leads to a lower HC_50_.

Further one-sided counterfactual analysis, presented in Figure 7b, provides an upper-bound explanation at the 90% confidence level for the hemolytic peptide dataset. Under otherwise unchanged conditions, when solvent accessibility exceeds −3.55, the probability that HC_50_ falls below roughly 160 μM reaches 90%. Moreover, when DDR_T is less than −2.59 or CeTD_VW1 is less than −2.61, the model estimates with 90% confidence that HC_50_ will be below approximately 110 μM.

To summarize, our results identify solvent accessibility, sequence transition ratio, and van der Waals volume distribution as key determinants in predicting hemolytic potential, linking hemolytic potential to residue accessibility, hydrophobic clustering, and hydrogen-bonding capacity [33,34,35]. These findings align with established hemolysis mechanisms, emphasizing the role of amino acid composition and spatial arrangement in peptide-membrane disruption. Furthermore, we demonstrate that counterfactual analysis offers conditional, causal insights beyond single-instance explanations, providing a solid modeling basis for elucidating peptide hemolysis mechanisms and guiding sequence optimization.

### 3.6. Server Implementation

**Figure 8:**
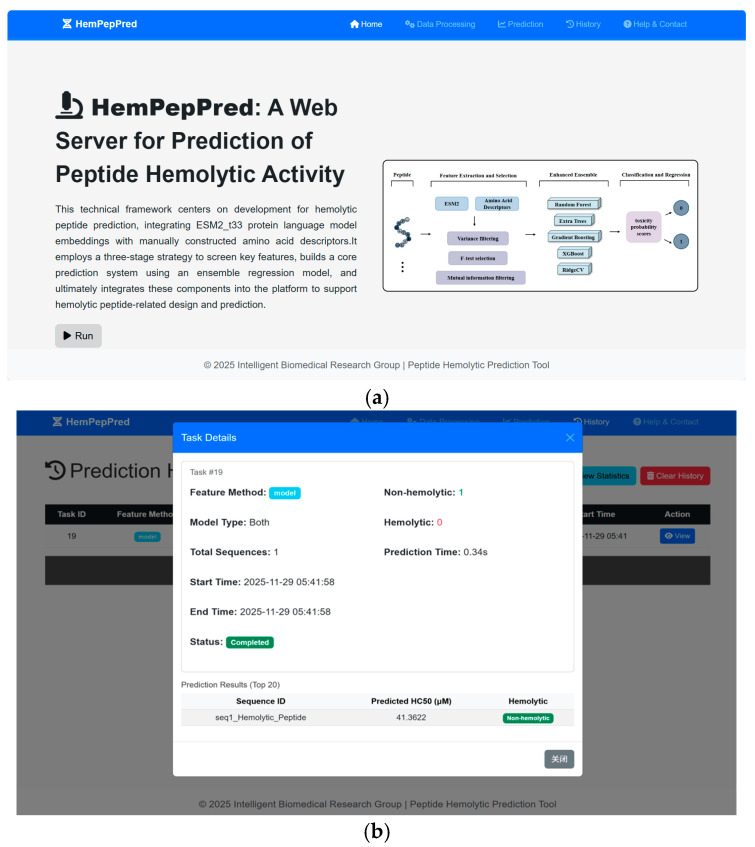
An overview of the end-to-end web platform with a user-friendly interface. (**a**) The main page of the web server. (**b**) The resultant output can be downloaded for further analysis.

Building upon our ensemble learning framework, we developed a web server (HemPepPred) for high-throughput peptide toxicity prediction, available at http://hem.cqudfbp.net (accessed on 22 October 2025). Users can submit sequence data on the main page (Figure 8a) and download the resulting outputs for further analysis (Figure 8b). The platform supports batch processing and automates the entire analytical pipeline—from sequence validation and statistical summarization to feature extraction, model prediction, and report generation.

To efficiently process large-scale datasets, the feature extraction module integrates ESM2_t33 embeddings with traditional AAD, leveraging batch parallelization to accelerate computation. Powered by PyTorch (2.3.1), the backend prediction engine employs an ensemble architecture that supports both classification and regression tasks, outputting toxicity probabilities and predicted labels. The web frontend, developed using Flask, HTML, and CSS, provides an intuitive interface for user interaction, while integration with MySQL ensures reliable data management and operational stability.

## 4. Conclusions

This study introduces an ensemble-learning regression framework for the quantitative prediction of peptide hemolytic activity by integrating amino acid descriptors (AADs) with protein language model embeddings. The proposed ensemble model outperforms conventional baselines, demonstrating that engineered descriptors and language model embeddings contribute complementary information. A multi-stage feature selection strategy further enhances predictive accuracy and generalization. Interpretability analyses identified CeTD descriptors as key determinants, providing mechanistic insights into hemolysis.

While the current framework achieves promising predictive performance, it remains limited by the scale and diversity of available experimental data, as well as by its reliance on sequence-derived features. These constraints may affect the model’s generalizability to novel peptide classes or varying experimental conditions. Future extensions could involve integrating 3D structural descriptors, physicochemical simulations, or transfer learning from broader peptide datasets to improve robustness, interpretability, and applicability.

The proposed framework paves the way for more accurate and interpretable prediction of peptide toxicity, facilitating safer peptide design and practical applications in biotechnology.

## Figures and Tables

**Table 1:** Sample Statistics of the Hemopi2 Dataset.

Dataset	Training Dataset		Test Dataset	
	Positive	Negative	Positive	Negative
Taken from (Rathore et al. [28])	713	828	178	207
Taken from (Karasev et al. [17])	826			

**Table 2:** Ablation study results based on the ensemble learning model.

Method	Feature (dim)	MAE	RMSE	R^2^	R
Without AAD	Without feature selection (1024 + 1280 = 2304)	0.3689	0.4779	0.4364	0.6772
	Feature selection	0.3471	0.4528	0.4942	0.7173
Without ESM2_t33	Without feature selection (1024 + 1167 = 2191)	0.3636	0.4729	0.4482	0.6753
	Feature selection	0.3115	0.4201	0.5645	0.7527
Without ProtT5	Without feature selection (1167 + 1280 = 2447)	0.3249	0.4271	0.55	0.7494
	Feature selection (633)	0.3130	0.4200	0.57	0.7551
Fusion of three features	Without feature selection (3471)	0.3377	0.4448	0.5118	0.7250
	Feature selection	0.3191	0.4224	0.5600	0.7520

**Table 3:** Performance comparison across different machine learning models (dim = 633).

Regressor	MAE	RMSE	R^2^	R
RF	0.326	0.437	0.529	0.732
XGB	0.334	0.453	0.494	0.708
DT	0.330	0.443	0.185	0.442
ADB	0.254	0.400	0.374	0.625
SVM	0.459	0.594	0.131	0.439
KNN	0.368	0.50	0.388	0.623
ET	0.306	0.420	0.560	0.751
Ours	0.313	0.420	0.565	0.755

**Table 4:** A randomly selected peptide sequence prediction from the test set.

Sequence	True_ HC_50_	Predicted_ HC_50_	True_ Hemolytic	Predicted_ Hemoplytic	Log_ True	Log_ Predicted
GIMSSLMKKLKAHIAK	400.0	300.9	1	1	2.60	2.48

## Data Availability

The original contributions presented in this study are included in the article/Supplementary Material. Further inquiries can be directed to the corresponding author.

## Supplementary Materials

The following supporting information can be downloaded at: https://www.mdpi.com/article/10.3390/foods14234143/s1, Table S1: Hyperparameter selection for ensemble learning.
