# Supplementary material for: HemPepPred: Quantitative Prediction of Peptide Hemolytic Activity Based on Machine Learning and Protein Language Model–Derived Features
Source: Foods. 2025 Dec 3;14(23):4143. doi: 10.3390/foods14234143 (PMC12692575; doi:10.3390/foods14234143)
Supplement: Supplementary file 1 [file foods-14-04143-s001.zip › foods-3971948-Supplementary Material.pdf]

Supplementary Material

Hemolytic Peptide Prediction via Feature Fusion and Ensemble Learning with Web Platform Implementation

Xiang Li †, Wanting Zhao †, Xiao Liang, Xinlan Zhuo, Shuang Yu and Guizhao Liang *

Key Laboratory of Biorheological Science and Technology, Ministry of Education, College of Bioengineering, Chongqing University, Chongqing 400044, China; 202319021049t@stu.cqu.edu.cn (X.L.); 202419021001@stu.cqu.edu.cn (W.Z.); 20241901014@stu.cqu.edu.cn (X.L.); 202319131087t@stu.cqu.edu.cn (X.Z.); 202319131159@stu.cqu.edu.cn (S.Y.)

* Correspondence: gzliang@cqu.edu.cn; Tel.: +86-23-65102507

† These authors contributed equally to this work.

Table S1. Hyperparameter selection for ensemble learning

Model	Hyperparameter name	Default value	Range
Feature selection	target_features	800	400 ~ 1200<br>（Step size 200）
	variance_threshold	0.005	0.001 ~ 0.01<br>（Step size 0.002）
	k_f_ratio	0.8	0.6 ~ 1.0<br>（Step size 0.1）
	k_mi_ratio	0.5	0.3 ~ 0.7<br>（Step size 0.1）
Random forest	rf_n_estimators	100	50 ~ 300<br>（Step size 50）
	rf_max_depth	12	6 ~ 20<br>（Step size 2）
	rf_min_samples_split	8	2 ~ 16<br>（Step size 2）
	rf_min_samples_leaf	3	1 ~ 8<br>（Step size 1）
Extra Trees	et_n_estimators	100	50 ~ 300<br>（Step size 50）
	et_max_depth	10	5 ~ 18<br>（Step size 1）
	et_min_samples_split	8	2 ~ 16<br>（Step size 2）
	et_min_samples_leaf	3	1 ~ 8<br>（Step size 1）
Gradient boosting	gb_n_estimators	100	50 ~ 200<br>（Step size 25）
	gb_learning_rate	0.08	0.01 ~ 0.2<br>（Step size 0.01）

	gb_max_depth	6	3 ~ 12（Step size 1）
	gb_min_samples_split	8	2 ~ 16（Step size 2）
XGBoost	xgb_n_estimators	100	50 ~ 200（Step size 25）
	xgb_learning_rate	0.08	0.01 ~ 0.2（Step size 0.01）
	xgb_max_depth	6	3 ~ 10（Step size 1）
	xgb_subsample	0.8	0.6 ~ 1.0（Step size 0.1）
	xgb_colsample_bytree	0.8	0.6 ~ 1.0（Step size 0.1）
	xgb_gamma	0	0 ~ 5（Step size 0.5）
	xgb_reg_alpha	0	0 ~ 10（Step size 1）
	xgb_reg_lambda	1	0 ~ 10（Step size 1）
Ridge Regression	alphas	[0.1,1,10,100]	[0.01,0.1,1,10,100,1000]
	max_iter	1000	500 ~ 2000（Step size 250）
Data preprocessing	n_quantiles	min(1000, len(y))	200 ~ 1000（Step size 200）
